# Tumor-suppressive function of long noncoding RNA MALAT1 in glioma cells by downregulation of MMP2 and inactivation of ERK/MAPK signaling

**DOI:** 10.1038/cddis.2015.407

**Published:** 2016-03-03

**Authors:** Y Han, Z Wu, T Wu, Y Huang, Z Cheng, X Li, T Sun, X Xie, Y Zhou, Z Du

**Affiliations:** 1 Neurosurgery & Brain and Nerve Research Laboratory, The First Affiliated Hospital of Soochow University, Suzhou, Jiangsu, PR China

## Abstract

Metastasis-associated lung adenocarcinoma transcript 1 (MALAT1) is a type of long noncoding RNA. It is associated with metastasis and is a favorable prognostic factor for lung cancer. Recent studies have shown that MALAT1 plays an important role in other malignancies. But, little is known about the role of MALAT1 in glioma. In this study, quantitative reverse transcription PCR (qRT-PCR) was used to demonstrate that the expression of MALAT1 was lower than that in normal brain tissues. Stable RNA interference-mediated knockdown of MALAT1 in human glioma cell lines (U87 and U251) significantly promoted the invasion and proliferation of the glioma cells by *in vitro* assays. Conversely, overexpression of MALAT1 caused significant reduction in cell proliferation and invasion *in vitro*, and tumorigenicity in both subcutaneous and intracranial human glioma xenograft models. Furthermore, MALAT1-mediated tumor suppression in glioma cells may be via reduction of extracellular signal-regulated kinase/mitogen-activated protein kinase (ERK/MAPK) signaling activity and expression of matrix metalloproteinase 2 (MMP2). In conclusion, overall data demonstrated the tumor-suppressive role of MALAT1 in glioma by attenuating ERK/MAPK-mediated growth and MMP2-mediated invasiveness.

Glioma is one of the most common types of primary brain tumors in adults, and represents one of the most aggressive and lethal human cancer types.^1^ Despite some advances in early detection, most of the patients are at advanced stages at the time of diagnosis, and the prognosis of these patients still remains poor.^2^ Although intensive research has been performed to detect and validate a number of molecules associated with glioma cell invasion and cell proliferation, only a few molecular mechanisms have been revealed and translated into clinical application so far.

Long noncoding RNAs (lncRNAs) are types of transcriptional products of the eukaryotic genome comprising >200 nt in length.^3, 4^ In recent years, some lncRNAs have been found to be involved in carcinogenesis and cancer progression. Metastasis-associated lung adenocarcinoma transcript 1 (MALAT1) is one of the first found cancer-associated lncRNAs, and is also referred to as nuclear-enriched abundant transcript 2 (NEAT2).^5^ MALAT1 was initially found to be highly expressed in lung cancer and is a favorable prognostic factor for the survival of patients with stage I non-small-cell lung cancer (NSCLC).^6^ However, little is known about the role of MALAT1 in glioma progression. The prognosis of human glioma is poor, and the highly invasive nature of the disease represents a major impediment to current therapeutic modalities.^7^ At the molecular level, tumor cell invasion is mediated by a set of factors that initiate or promote cell motility, destruction of matrix, angiogenesis, and other biological events.^8, 9, 10, 11^ Extracellular signal-regulated kinase/mitogen-activated protein kinase (ERK/MAPK) signaling pathway mediates cell invasion and proliferation; aberrant activation of the ERK/MAPK signaling pathway has been observed in many types of human cancers including glioma.^12, 13, 14^ ERK/MAPK signaling orchestrates several key biological processes during the development and progression of cancer by inducing the transcription of a number of target genes regulating cell invasion. In particular, matrix metalloproteinase 9 (MMP9) and 2 (MMP2) levels increase with tumor progression in gliomas, and are thus known as key enzymes for invasion.^15^ In a previous study, it was found that MALAT1 promotes the proliferation and metastasis by activating the ERK/MAPK pathway.^16^ Hence this study investigated the expression of MALAT1 in glioma specimens and its regulation of glioma cell proliferation and invasion through the ERK/MAPK signaling pathway and expression/activation of MMP.

## Results

### Expression of MALAT1 in glioma

**Figure 1:**
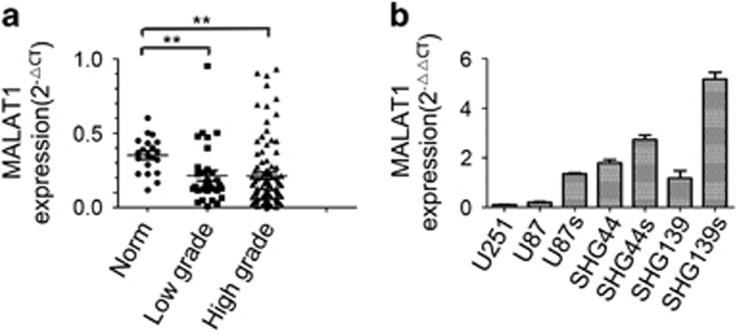
Expression of MALAT1 in glioma. (**a**) Expression of MALAT1 in human glioma tissues was lower than in noncancerous tissues (*P*<0.01). (**b**) Expression of MALAT1 in human glioma cell lines. Abbreviation: MALAT1, metastasis-associated lung adenocarcinoma transcript 1

To assess the expression of MALAT1 in noncancerous brain tissues and in different grades of glioma, the expression of MALAT1 was detected in 20 non-neoplastic brain tissues and in 132 human glioma tissue samples using quantitative reverse transcription PCR (qRT-PCR). Data showed that the expression of MALAT1 in noncancerous brain tissues was higher than in human brain glioma tissues (*P*<0.01), but there was no obvious difference between different degrees of malignancy in glioma (low-grade *versus* high-grade gliomas) (Figure 1a). To determine the effect of MALAT1 in glioma, seven glioma cell lines (including three subculture lines carrying features of neural stem-like cell) were examined. As shown in Figure 1b, glioma stem cell lines of U87, SHG44 and SHG139 expressed higher levels of MALAT1 than their parental lines. U251 expressed lower levels of MALAT1 than that of U87. U251 and U87 were used in the present study to show the effect of overexpression of MALAT1, in addition to shRNA-mediated knock down.

### Effects of MALAT1 shiRNA and overexpressing vector on the expression of MALAT1 in U87 and U251

**Figure 2:**
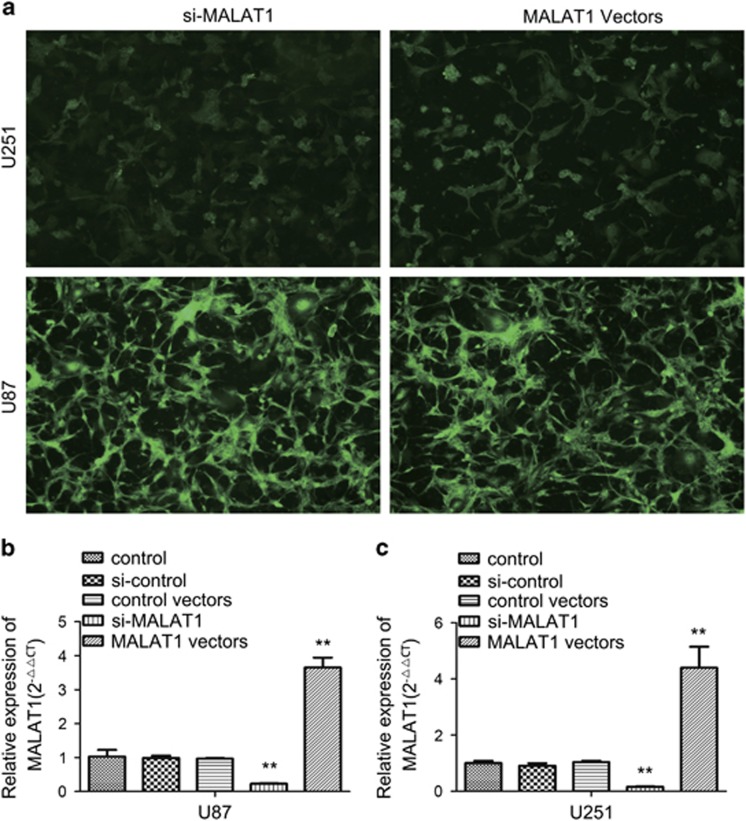
Effects of si-MALAT1- and MALAT1-overexpressing vectors on the expression of lncRNA MALAT1. (**a**) The transfection efficiency was determined 3 days after incubation with lentivirus at an MOI of 20. The transfected cells labeled with GFP were observed under a fluorescence microscope ( × 200). (**b** and **c**) Total RNA was extracted 4 days after infection, and the relative expression of MALAT1 was determined using quantitative real-time PCR. GAPDH was used as an internal control. The data represent the mean±S.D. of three independent experiments (**P<0.01). Abbreviations: GAPDH, glyceraldehyde-3-phosphate dehydrogenase; GFP, green fluorescence protein; lncRNA, long noncoding RNA; MALAT1, metastasis-associated lung adenocarcinoma transcript 1; MOI, multiplicity of infection; PCR, polymerase chain reaction; S.D., standard deviation

MALAT1 was lowly expressed in glioma tissues, hence the function of MALAT1 was investigated using siRNA and overexpression vector in two glioma cell lines (U87 and U251). The lentiviral transfection efficiencies of the U87 and U251 cells were determined by examining the expression of GFP under a microscope 72 h after transfection. The efficiency of lentiviral transfection in both of the U87 and U251 cells was higher than 90% (Figure 2a). The expression of MALAT1 in U87 and U251 cells was examined using real-time PCR analysis after transfection with lentivirus. The levels of expression of MALAT1 in the U87 and U251 cells transfected with MALAT1 overexpression vector were increased by 3.65-folds (*P*<0.01) and 4.40-folds (*P*<0.01), respectively, compared with the control cells (Figures 2b and c), while the levels of expression of MALAT1 in the U87 and U251 cells transfected with MALAT1-siRNA lentivirus (si-MALAT1) were decreased by 76.0% (*P*<0.01) and 74.9% (*P*<0.01), respectively, compared with the control cells (Figures 2b and c).

### MALAT1-suppressed U87 and U251 cell proliferation *in vitro*

**Figure 3:**
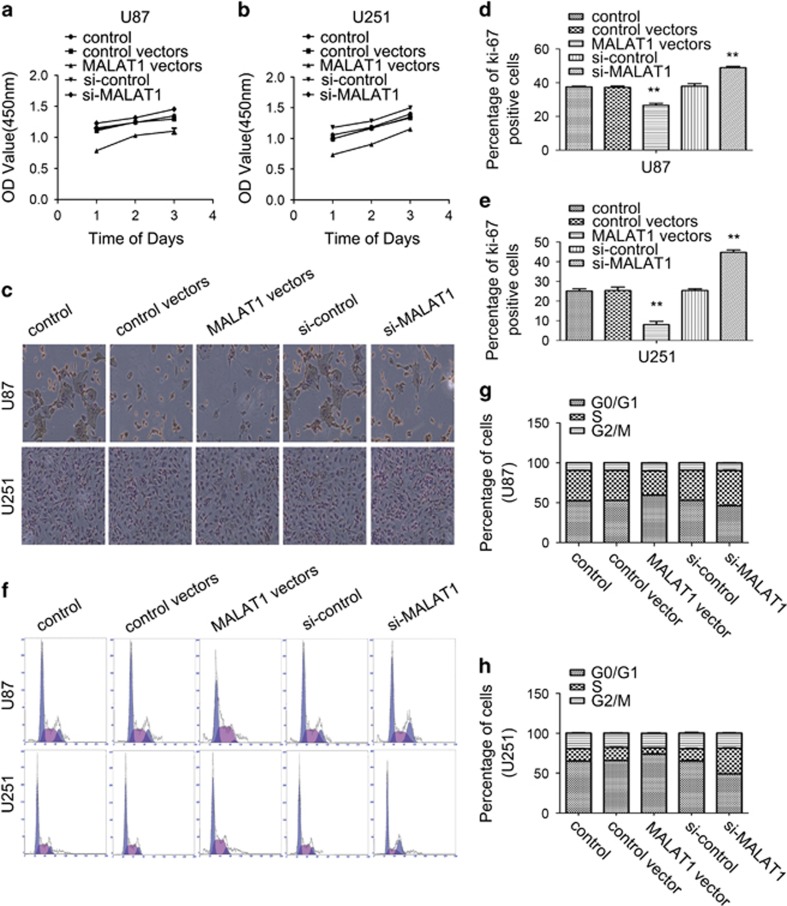
Effects of upregulation and downregulation of MALAT1 on U87 and U251 cell proliferation. (**a** and **b**) Cellular proliferation of untransfected or transfected U87 and U251 cells was measured using a CCK-8 assay daily for 3 days. (**c**) Cellular proliferation of untransfected or transfected U87 and U251 cells was measured by testing the expression of Ki-67. (**d** and **e**) The percentage of Ki-67-positive cells was calculated. Results are expressed as mean±S.D. from three independent experiments (*P*<0.01). (**f**) Untransfected or transfected U87 and U251 cells were stained by propidium iodide and analyzed using flow cytometry. (**g** and **h**) The percentage of cells in the G0/G1, S and G2/M phases of the cell cycle was calculated. Results are expressed as mean±S.D. from three independent experiments (*P*<0.01). Abbreviations: CCK-8, Cell Counting Kit-8; MALAT1, metastasis-associated lung adenocarcinoma transcript 1; S.D., standard deviation

To investigate whether the overexpression of MALAT1 could influence the proliferation of glioma cells *in vitro*, CCK-8 assay was performed. Figures 3a and b show that the proliferation abilities of the U87 and U251 cells decreased significantly after overexpression of MALAT1, but downregulation of MALAT1 increased the proliferation ability. Later the expression of Ki-67 by these glioma cells was tested. With the upregulation of MALAT1, the expression of Ki-67 decreased, while downregulation of MALAT1 increased the expression of Ki-67 (Figures 3c–e). To understand the effects of MALAT1 on cell-cycle distribution, glioma cells were analyzed using flow cytometry. The results indicated that overexpression of MALAT1 decreased the percentage of S cells and increased the percentage of G0/G1 cells (*P*<0.01, respectively), while downregulation of MALAT1 in cells showed the opposite results (Figures 3f–h).

### MALAT1-suppressed U87 and U251 cell proliferation *in vivo*

**Figure 4:**
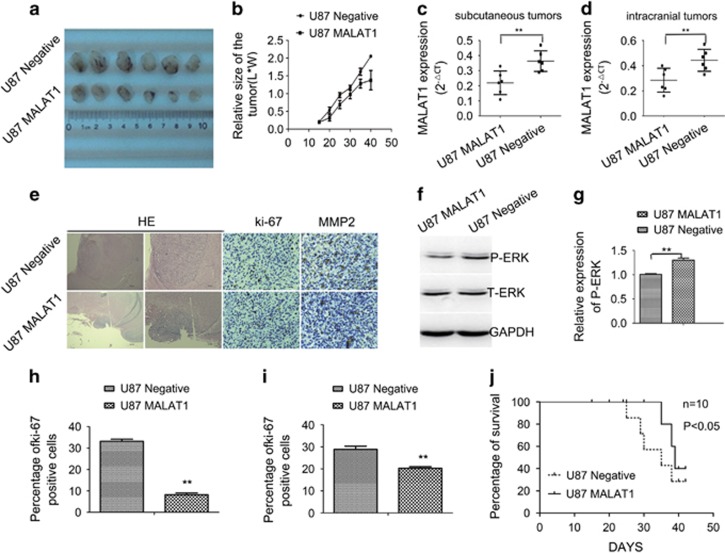
Effects of overexpression of MALAT1 on proliferation *in vivo*. (**a **and **b**) Overexpression of MALAT1 reduced the growth of glioma in a subcutaneous glioma nude mouse model. (**c** and **d**) MALAT1 expression in subcutaneous and incratranial tumors (*P*<0.01). (**e**) Immunohistochemistry showed that overexpression of MALAT1 reduced the expression of Ki-67 and MMP2 in intracranial xenograft of U87. (**f **and **g**) Western blotting showed that overexpression of MALAT1 reduced the expression of P-ERK (*P*<0.01). (**h **and **i**) The percentage of Ki-67- and MMP2-positive cells, respectively, calculated from densitometry immunohistochemistry signaling. Results are expressed as mean±S.D. from three independent experiments (*P*<0.01). (**j**) Kaplan–Meier survival curves for nude mice implanted intracranially with U87 and its MALAT1 transfectants. MALAT1, metastasis-associated lung adenocarcinoma transcript 1

To determine the effects of MALAT1 on glioma cell growth *in vivo*, MALAT1 overexpressed or control U87 cells were injected into the left axilla of nude mice. As shown in Figures 4a and b, the growth of tumors from the MALAT1-overexpressed xenografts of glioma cell line was significantly slower compared with that of tumors formed from the control cells (*P*<0.01).

To assess the expression of MALAT1 in subcutaneous tumors and intracranial tumors, the expression of MALAT1 was detected in tumors formed by U87-negative cells and U87 cells transfected with MALAT1 vectors. Data showed that the expression of MALAT1 in tumors formed by U87 cells transfected with MALAT1 vectors was higher than U87-negative cells (Figures 4c and d) (*P*<0.01).

The intracranial tumors were removed together with surrounding brain tissues prior to killing moribund mice normally shown 20–30% weight loss, fixed, embedded and sectioned for immunohistochemical analysis. As shown in Figures 4e, h and i, intracranial xenografts of U87 cells transduced to overexpress MALAT1 had reduced proliferation index by Ki-67 staining and invasive potential by MMP2 staining compared with empty vector control. To investigate whether the ERK/MAPK pathway was affected *in vivo*, total protein was extracted from the intracranial tumors, and the results of western blotting showed that levels of phosphorylated ERK1/2 decreased in tumors formed by U87 cells transfected with MALAT1 vectors (Figures 4f and g).

To further determine the effect of MALAT1 on glioma tumorigenicity in orthotopic location, MALAT1- or empty vector-infected U87 was implanted into nude mice frontal lobe of the brain. Overall survival data of mice demonstrated a significant effect of MALAT1 in suppressing the growth of U87-xenograft (Figure 4j).

### Effects of upregulation and downregulation of MALAT1 on U87 and U251 cell invasiveness

**Figure 5:**
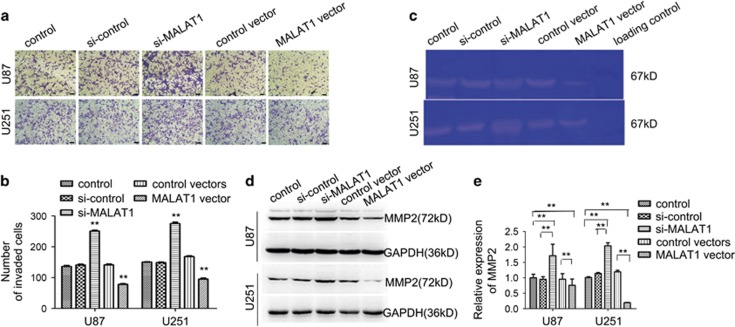
Effects of upregulation and downregulation of MALAT1 on glioma cell invasion *in vitro*. (**a** and **b**) The transwell invasion system showed that upregulation of MALAT1 decreased the invasive cell numbers and downregulation of MALAT1 increased the invasive cell numbers compared with the cultures transfected with the negative control oligonucleotide. Each bar represents mean±S.D. from three independent experiments (*P*<0.01). (**c**) Gelatin zymography assay showed that the overexpression of MALAT1 reduced the MMP2 protein and downregulation of MALAT1 increased the MMP2 protein secreted from cells. (**d **and **e**) The expression of MMP2 was obviously inhibited by the upregulation of MALAT1 and upregulated by the downregulation of MALAT1. Each bar represents mean±S.D. from six independent experiments (*P*<0.01). MALAT1, metastasis-associated lung adenocarcinoma transcript 1; MMP2, matrix metalloproteinase 2; S.D., standard deviation

To assess the effects of MALAT1 on the invasiveness of glioma cells, a transwell invasion system was used. The numbers of invasive cells with overexpressed MALAT1 were significantly reduced and the numbers of invasive cells with downregulated MALAT1 were increased compared with those of control cells (Figures 5a and b). To further explore the mechanism of MALAT1 in suppressing glioma cell invasion, MMP2 and TIMP3 were examined using western blotting. Data showed that the expression level of MMP2 was markedly reduced due to the overexpression of MALAT1 and that the opposite result was observed by the knockdown of MALAT1 (Figures 5d and 5e). Gelatin zymography assays showed a single clear band at ~67 kDa, indicating MMP2-mediated gelatin degradation, and this study confirmed similar changes at the protein level (Figure 5c). However, MALAT1 had little effect on the expression of TIMP3 (data not shown), further indicating that the mechanism of MALAT1-suppressed glioma cell invasion is via downregulation of expression of MMP2 at the protein level.

### Suppression of MAPK kinase pathways by overexpression of MALAT1

**Figure 6:**
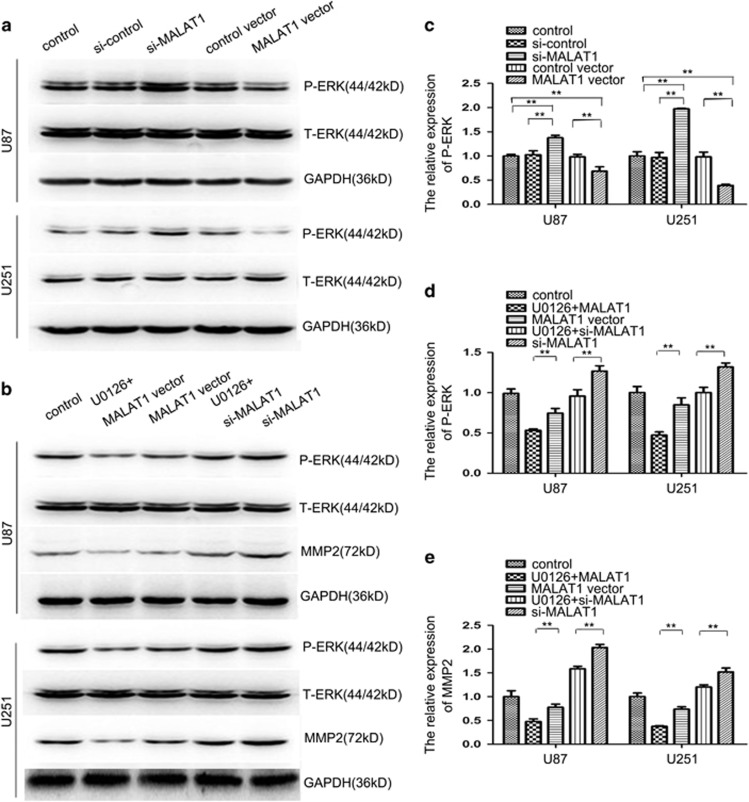
MALAT1 inactivated the ERK/MAPK pathway in the glioma cell lines. (**a** and **c**) Western blot analysis showed that upregulation of MALAT1 reduced the expression of P-ERK and downregulation of MALAT1 increased its expression. Each bar represents the mean±S.D. from three independent experiments (**P<0.01). (**b**, **d** and **e**) Western blot analysis showed that inhibition of ERK/MAPK signaling abrogated the downregulation of MALAT1-induced expression of P-ERK and MMP9 and promoted the effects of upregulation of MALAT1. Each bar represents the mean±S.D. from three independent experiments (**P<0.01). ERK/MAPK, extracellular signal-regulated kinase/mitogen-activated protein kinase; MALAT1, metastasis-associated lung adenocarcinoma transcript 1; MMP, matrix metalloproteinase; S.D., standard deviation

To determine the possible mechanism by which MALAT1 regulated the proliferation of glioma cells, western blot analysis was performed to investigate the effects of knockdown and overexpression of MALAT1 on the ERK/MAPK pathway, which is often aberrantly activated in human cancers and contributes to enhanced cell proliferation and invasiveness. Western blot analysis showed that downregulation of MALAT1 significantly increased the levels of phosphorylated ERK1/2, and upregulation of MALAT1 reduced the levels of phosphorylated ERK1/2, while no detectable changes were observed in the total levels of ERK1/2 (Figures 6a and c). To further verify the role of signaling activation and inactivation in MALAT1-aberrant expressed glioma cells, the addition of U0126 (a specific inhibitor of MEK/ERK) was used to pretreat cells. Results showed that with the presence of U0126, the upregulation of phosphorylated ERK1/2 and MMP2 by the knockdown of MALAT1 was attenuated. Consistently, the downregulation of phosphorylated ERK1/2 and MMP2 by the overexpression of MALAT1 was enhanced (Figures 6b, d and e). These results indicate that MALAT1 regulates the ERK/MAPK signaling activity, which regulates MMP2 and overall glioma cell proliferation and invasion.

## Discussion

Invasion and spread of solid gliomas are the major causes of death in patients with glioma.^17^ Therefore, identification of novel methods that can effectively inhibit the growth and invasion of gliomas is needed. The present study showed that lncRNA MALAT1 has a tumor-suppressive function in glioma via suppressing both growth and invasion of cells, which is consistent with its lower expression levels in gliomas compared with normal brain tissues.

lncRNAs were initially considered to be spurious transcriptional noise, but recent evidence suggests that they may play a major biological role in cellular development and human diseases.^18, 19^ lncRNAs such as HOTAIR (HOX antisense intergenic RNA), ANRIL (antisense noncoding RNA (ncRNA) in the INK4 locus), CUDR (cancer upregulated drug resistant) and MVIH (lncRNA associated with microvascular invasion in hepatocellular carcinoma (HCC)) have been shown to act as key molecules in the regulation of processes such as chromatin remodeling, transcription and post-transcriptional processing.^20, 21^ MALAT1, which is also known as HCN, NEAT2, PRO2853 and NCRNA00 047, is located at chromosome 11q13.1 and encodes a polyadenylated ncRNA of ~8 kb.^6, 22^ Ji *et al.* found that MALAT1 was overexpressed in early-stage metastasizing NSCLC. High expression of MALAT1 was considered to be correlated with poor prognosis in patients with NSCLC.^6^ Therefore, MALAT1 has been proposed as a prognostic marker for metastasis and survival of patients with NSCLC. Additionally, Xie *et al.*^23^ showed that MALAT1 was highly expressed in human nasopharyngeal carcinoma cell lines and can enhance the proliferation, invasion and metastasis of CNE-1 cells. Lai *et al.*^24^ found that MALAT1 is an independent prognostic factor for the recurrence of HCC after liver transplantation.

MALAT1 is commonly located in nuclear speckles.^25^ Nuclear speckle is an important subnuclear structure containing a large amount of nuclear proteins involved in precursor messenger mRNA alternative splicing and RNA transportation.^26^ This evidence supports the hypothesis that MALAT1 could be a regulator of post-transcriptional RNA processing or modification. Moreover, MALAT1 was shown to promote the invasion of cancer cell by inducing the expression of MMP9, and the activation of the ERK/MAPK pathway participates in this process.^16^ In addition, MALAT1 can also interact with the unmethylated form of CBX4, which controls the relocation of growth-control genes between the polycomb bodies and interchromatin granules, sites of silent or active gene expression, respectively.^27^

To elucidate the possible mechanism by which MALAT1 regulates the proliferation and invasion of glioma cell, western blot analysis of the key molecular factors of cancer-related pathways, such as nuclear factor kappaB, mTOR, Akt and others (data not shown), was performed.^28, 29, 30^ The ERK/MAPK pathway is one of the most important signal transduction pathways, and upregulation of MALAT1 inhibits the growth and invasion of tumor by inactivating this signaling cascade. It was observed that in U87 and U251 cells, overexpression of MALAT1significantly reduced the expression of phosphorylated ERK1/2. However, no detectable changes in the expression of total ERK1/2 protein were observed. To further verify the role of signaling activation and inactivation in MALAT1-aberrant expressed glioma cells, the addition of U0126 (a specific inhibitor of MEK/ERK) was used to pretreat cells. Results showed that inhibition of ERK1/2 signaling suppressed MALAT1 low expression-induced levels of phosphorylated ERK1/2 and MMP2.

The results of the present study were a little different from others. MALAT1 was shown to act as a tumor promoter gene in gallbladder cancer, lung cancer, colorectal cancer, etc.^31, 32, 33, 34^ But in the present study, it was proved that MALAT1 acts as a tumor suppressor gene. It may be caused by the different types of tumors. And the direct link between the ERK/MAPK pathway and MALAT1 remains unclear. In previous studies, it has been proved that knockout of MALAT1 in lung cancer cells could significantly reduce the expression of several metastasis-related genes including Glypican 6 (GPC6) and C-X-C motif chemokine 5 (CXCL5),^35, 36^ while depletion of GPC6 or CXCL5 could lead to the inactivation of the MAPK pathway. Hence it was hypothesized that MALAT1 could probably inactivate the ERK/MAPK pathway via regulation of the expression of GPC6 or CXCL5 genes in glioma, which requires further study Overall data suggest tumor-suppressive effect of MALAT1 in glioma, by inhibiting the proliferation and invasion of glioma, and inactivates via regulation of the ERK/MAPK pathway and expression of MMP2.

## Materials and Methods

### Human tissue samples

For the study, 132 glioma samples were obtained from 132 Chinese patients (83 men and 49 women) between March 2011 and September 2013 from the Department of Neurosurgery, Brain and Nerve Research Laboratory of The First Affiliated Hospital of Soochow University. Two patients had grade I (pilocytic astrocytoma), 30 patients had grade II (diffuse astrocytoma), 60 patients had grade III (anaplastic astrocytoma) and 40 patients had grade IV (primary brain glioblastoma), according to the 2007 World Health Organization's classification system. The mean ages of the patients at the time of surgery were 49 years for men and 48.1 years for women. Twenty non-neoplastic brain tissue samples were obtained from adult patients with craniocerebral injuries, which required partial resections of brain tissue as decompression treatment to reduce intracranial pressure. All human samples were used in accordance with the policies of the institutional review board of The First Affiliated Hospital of Soochow University.

### Cell cultures

The human U87 and U251 cell lines were purchased from the Cell Bank Type Culture Collection of the Chinese Academy of Sciences (Shanghai, China). Cells were grown in Dulbecco's modified Eagle's medium (DMEM) (Hyclone; Thermo Fisher Scientific, Waltham, MA, USA) supplemented with 10% fetal bovine serum (FBS) (GIBCO, Invitrogen Inc., Carlsbad, CA, USA).

### Lentivirus-mediated RNA interference

The following short hairpin RNA (shRNA) 19 was used to target human MALAT1: sense: 5′-TGCTGTGTACTATCCCATCACTGAAGGTTTTGGCCACTGACTGACCTTCAGTGGGGATAGTACA-3′ antisense: 5′-CCTGTGTACTATCCCCACTGAAGGTCAGTCAGTGGCCAAAACCTTCAGTGATGGGATAGTACAC-3′.

The sequence of the negative control shRNA was Negative-F: tgctgAAATGTACTGCGCGTGGAGACGTTTTGGCCACTGACTGACGTCTCCACGCAGTACATTT; Negative-R: cctgAAATGTACTGCGTGGAGACGTCAGTCAGTGGCCAAAACGTCTCCACGCGCAGTACATTTC.

These shRNAs were synthesized and inserted into the pFH1UGW lentivirus core vector containing a cytomegalovirus (CMV)-driven enhanced green fluorescent protein reporter gene; expression of the shRNA was driven by the H1 promoter. Recombinant lentivirus-expressing MALAT1-siRNA or control siRNA (si-MALAT1 or si-Control) was produced by Invitrogen Inc.

### Lentiviral constructs

The sequence of MALAT1 was synthesized and subcloned into lentivector-transferred plasmid pCDH-CMV-MCS-EF1-coGFP to generate pCDH-CMV-MALAT1-EF1-coGFP. The recombinant vector pCDH-CMV-MALAT1-EF1-coGFP or the control vector pCDH-CMVMCS-EF1-coGFP was triple transfected with the packaging vectors psPAX2 and pMD2.G into 293T cells using calcium chloride to produce the lentivirus by Invitrogen Inc. Glioma cells were infected with control or MALAT1-expressing lentivirus. The expression of MALAT1 in cells was determined using quantitative PCR.

### Inhibition of expression of P-ERK1/2 by U0126 in cells

U87 and U251 cells were seeded onto a six-well plate and incubated at 37 °C overnight. The cells were then treated with 10 mM U0126 (Beyotime, Shanghai, China), a MAPK/ERK kinase inhibitor that inhibits MEK1/2 for downregulation of expression of P-ERK for 72 h. Then, the cells were infected with lentivirus.

### Quantitative reverse transcription PCR

Total RNA from tissues and cells was isolated using TRIzol reagent (Invitrogen Inc.). Relative levels of MALAT1 lncRNA were examined using SYBRgreen real-time quantitative reverse transcription PCR (qRT-PCR) (Applied LightCycler480) and were normalized to levels of glyceraldehyde-3-phosphate dehydrogenase (GAPDH) mRNA. The following primers were used: lncRNA MALAT1 forward primer 5′-CTAAGGTCAAGAGAAGTGTCAG-3′ reverse primer 5′-AAGACCTCGACACCATCGTTAC-3′. GAPDH forward primer 5′-AACGGATTTGGTCGTATTG-3′ reverse primer 5′-GGAAGATGGTGATGGGATT-3′. Relative expression was calculated using the 2^−△△CT^ method. All qRT-PCR analyses were performed in triplicate, and the data are presented as means±standard errors of the means.

### Cell-cycle analysis

Both nontransfected and transfected U87 and U251 cells were harvested by trypsinization, washed three times in cold PBS and fixed in 70% ethanol at 4 °C overnight. After fixation, the cells were washed and resuspended in cold PBS and incubated in a solution of 10 mg/ml RNase and 1 mg/ml propidium iodide (Sigma-Aldrich, St. Louis, MO, USA) at 37 °C for 30 min in the dark. Finally, the DNA content was determined using flow cytometry (BD Biosciences, Franklin Lake, NJ, USA). The percentage of cells in the G0/G1, S and G2/M phases was determined using Cell Quest acquisition software (BD Biosciences).

### Cell proliferation assay

Cell proliferation was quantified using the Cell Counting Kit-8 (CCK-8; Beyotime). Briefly, 100 *μ*l of cells from the five groups (control, vector control, vector-MALAT1, si-control and si-MALAT1) were seeded onto a 96-well plate at a concentration of 2000 cells per well and were incubated at 37 °C. At daily intervals (days 1, 2 and 3), the optical density was measured at 450 nm using a microtiter plate reader, and the rate of cell survival was expressed as the absorbance. The results represent the mean of six replicates under the same conditions.

### Tumor cell matrigel invasion assay

Overall, 5 × 10^5^ cells (U87 and U251) from each group were transferred on the top of Matrigel-coated invasion chambers (24-well insert, 8 *μ*m pore size; BD Biosciences) in serum-free DMEM. DMEM containing 0.05% FBS was added to the lower chamber as a chemoattractant. After an incubation period of 48 h, noninvading cells were removed from the inner part of the insert using a cotton swab. Cells on the lower membrane surface were fixed in 4% formaldehyde and stained with 0.1% crystal violet. Invading cells were manually counted in five randomly chosen fields under a microscope, and photographs were taken.

### Gelatin zymography

Equal numbers of glioblastoma cells/transfectants were plated in DMEM/F12 containing 5% calf serum overnight to reach 80% confluence. Later culture medium was replaced with serum-free medium following rinsing of cells with PBS, and culture was continued for 48 h. The conditioned medium was collected after removal of floating cells. Proteins in the medium were precipitated with four volumes of cold acetone, spun immediately at 14  000 r.p.m. for 5 min at 4 °C, and resuspended in radioimmunoprecipitation assay buffer containing 1 × Protease Inhibitor Cocktail. Gelatin zymography was performed using equal amounts of protein (0.5–1 *μ*g for U87 and 2–4 *μ*g for U251). Protein readings were based on the control for correct protein loading, and each experimental sample was repeated two to three times to verify the results.

### Western blot analysis

The primary antibodies used were anti-MMP2 (Abcam, Tokyo, Japan), anti-TIMP3 (Abcam), anti-P-ERK1/2 and anti-T-ERK1/2 (Abcam). Protein samples were separated with 12% sodium dodecyl sulfate-polyacrylamide gel electrophoresis and transferred onto nitrocellulose membranes. Membranes were incubated with primary antibodies overnight at 4 °C. Membranes were washed and incubated for 2 h with horseradish peroxidase (HRP)-conjugated anti-rabbit secondary antibodies (Prosci Inc., Poway, CA, USA), followed by detection and visualization using electrochemiluminescence western blotting detection reagents (Pierce antibodies; Thermo Fisher Scientific).

### Immunohistochemistry

Formalin-fixed paraffin-embedded U87 tumors were cut with a microtome into 6-*μ*m sections. Antigen retrieval was performed in 10 mM sodium citrate buffer of pH 6 for 16 min at 96–98 °C. Slides were incubated with primary antibodies against Ki-67 (Boster Bioengineering Co., Wuhan, China), and with antibodies against MMP2 and TIMP3 (Abcam). Sections were subsequently incubated with the Cell & Tissue Staining Kit HRP-DAB system (R&D Systems, Minneapolis, MN, USA), according to the manufacturer's instructions. Immunostaining was performed with known positive and negative tumor controls and were blindly evaluated by a pathologist.

### Subcutaneous and intracranial implanted models

To explore the effects of MALAT1 on tumor growth and invasion *in vivo*, an intracranial and a subcutaneous nude mouse model was established using 1 × 10^5^ and 1 × 10^6^ U87, respectively, cells infected with lentivirus-expressing MALAT1 or empty vector. On day 17 postimplantation, caliper measurements were performed to assess the growth of tumor. And animals with cells injected intracranially into the right frontal lobes of 4–6-week-old nu/nu mice brains were observed daily for neurologic symptoms. Those that were moribund were killed and that date was used to calculate survival.

### Statistical analysis

Statistical analyses were performed using the SPSS software, Version 13.0 (SPSS, Chicago, IL, USA). Statistical significance was determined using two-tailed Student's *t-*test. A *P-*value of less than 0.05 was considered statistically significant.

## Abbreviations

MALAT1: metastasis-associated lung adenocarcinoma transcript 1
qRT-PCR: quantitative reverse transcription PCR
ERK/MAPK: extracellular signal-regulated kinase/mitogen-activated protein kinase
MMP2: matrix metalloproteinase 2
MMP9: matrix metalloproteinase 9
lncRNAs: long noncoding RNAs
NEAT2: nuclear-enriched abundant transcript 2
NSCLC: non-small-cell lung cancer
DMEM: Dulbecco's modified Eagle's medium
FBS: fetal bovine serum
shRNA: short hairpin RNA
CMV: cytomegalovirus
GAPDH: glyceraldehyde-3-phosphate dehydrogenase
CCK-8: Cell Counting Kit-8
HRP: horseradish peroxidase
HOTAIR: HOX antisense intergenic RNA
CUDR: cancer upregulated drug resistant
HCC: hepatocellular carcinoma
GPC6: Glypican 6
CXCL5: C-X-C motif chemokine 5
